# ElTetrado: a tool for identification and classification of tetrads and quadruplexes

**DOI:** 10.1186/s12859-020-3385-1

**Published:** 2020-01-31

**Authors:** Tomasz Zok, Mariusz Popenda, Marta Szachniuk

**Affiliations:** 1 Institute of Computing Science and European Centre for Bioinformatics and Genomics, Poznan University of Technology, Piotrowo 2, Poznan, 60-965 Poland; 2 Poznan Supercomputing and Networking Center, Jana Pawla II 10, Poznan, 61-139 Poland; 3 Institute of Bioorganic Chemistry, Polish Academy of Sciences, Noskowskiego 12/14, Poznan, 61-704 Poland

**Keywords:** Quadruplexes, Classification, Secondary structure topology, Dot-bracket notation, Arc diagram

## Abstract

**Background:**

Quadruplexes are specific structure motifs occurring, e.g., in telomeres and transcriptional regulatory regions. Recent discoveries confirmed their importance in biomedicine and led to an intensified examination of their properties. So far, the study of these motifs has focused mainly on the sequence and the tertiary structure, and concerned canonical structures only. Whereas, more and more non-canonical quadruplex motifs are being discovered.

**Results:**

Here, we present ElTetrado, a software that identifies quadruplexes (composed of guanine- and other nucleobase-containing tetrads) in nucleic acid structures and classifies them according to the recently introduced ONZ taxonomy. The categorization is based on the secondary structure topology of quadruplexes and their component tetrads. It supports the analysis of canonical and non-canonical motifs. Besides the class recognition, ElTetrado prepares a dot-bracket and graphical representations of the secondary structure, which reflect the specificity of the quadruplex’s structure topology. It is implemented as a freely available, standalone application, available at https://github.com/tzok/eltetrado.

**Conclusions:**

The proposed software tool allows to identify and classify tetrads and quadruplexes based on the topology of their secondary structures. It complements existing approaches focusing on the sequence and 3D structure.

## Background

One of the key problems that can be solved by structural bioinformatics is the discovery and analysis of motifs that form in molecular structures. Recurring patterns are searched for at all levels of the architecture: in sequence, secondary and tertiary structure [1–8]. Motif’s discovery entails the research into its influence on the molecule functions. Among the motifs that are currently being researched in many areas of life sciences are quadruplexes. These structures were first found in DNA, in the late 1960s. However, only after their association with targeted therapies against cancer and neurodegenerative diseases, the study on these motifs started to expand [9]. Today, computationally-supported analysis of quadruplex characteristics constitutes a relatively new challenge in structural bioinformatics and computational biology [10, 11].

The building unit of the quadruplex is a tetrad (called also a quartet). Single tetrad is made up of four nucleotide residues arranged in a plane. Each of them forms two non-canonical bonds (one along the Watson-Crick edge, and one along the Hoogsteen edge) with the others. If at least two tetrads are stacked upon one another at a distance of about 3.3 Å, the quadruplex is created [12]. Its structure is considered at the sequence level, examining the N-tracts which are part of the motif. The 3D structure analysis encompasses the properties of tetrads, stems and loops that connect external quartets. The structural diversity of quadruplexes is large and new motifs are constantly being discovered [13–18]. Meanwhile, most studies have been limited to their specific subsets. In particular, many researchers focused on canonical quadruplexes built of Guanines (so-called G4s). The latter ones were the subject of analysis and formalism proposed in [19].

Recently, we have proposed a new approach to the analysis and classification of tetrads and quadruplexes [20]. Its foundation is the secondary structure topology of these motifs. Our concept has resulted from the observation of patterns (O-shaped, N-shaped, and Z-shaped tracks) occurring in the secondary structure diagrams of quadruplexes generated and annotated by RNApdbee 2.0, a web server from the RNApolis toolset [21, 22]. We have defined six classes of tetrad topologies (so called ONZ taxonomy) that correspond to pairings between quartet-forming nucleotides. Next, we have introduced ONZ-based formalism for quadruplexes [20]. It addresses both canonical and non-canonical motifs. Finally, we have designed two-line dot-bracket that allows for unambiguous encoding of the secondary structure of tetrads and quadruplexes, and we have adjusted arc diagrams to represent a variety of their topologies.

Here, we present ElTetrado, a software tool that accompanies our novel classification approach, and the results of its application in processing nucleic acids structures from Protein Data Bank. ElTetrado identifies tetrads and quadruplexes in nucleic acid structures and assigns them to categories according to ONZ taxonomy. It also provides dot-bracket encoded secondary structures of these motifs and the corresponding arc diagrams. The application is freely available at https://github.com/tzok/eltetrado.

## Results

In the preliminary computational experiments with ElTetrado, we aimed to find the distribution of tetrads and quadruplexes in different ONZ categories [20]. We focused on unimolecular motifs, as the classification is based on the order of nucleotides in the chain, which is unambiguously determined only in the case of single-stranded structures. The input dataset was constructed from PDB-deposited nucleic acid structures on 18 April, 2019. The research showed that in unimolecular structures O-type tetrads constituted the majority (75%). We have assumed that a similar situation should occur in bi- and tetramolecular motifs.

This assumption has become the starting point for the development of a procedure for the classification of tetrads and quadruplexes made up of more than one strand. The procedure optimizes the processing order of the motif’s strands so as to maximize the allocation of tetrads to the O category. The result of its application is the assignment of the ONZ categories to bi- and tetramolecular tetrads and quadruplexes as well. Strand reordering can be switched off by calling ElTetrado with --no-reorder parameter. In such a program call, bi- and tetramolecular motifs are not classified according to the ONZ taxonomy.

**Fig. 1:**
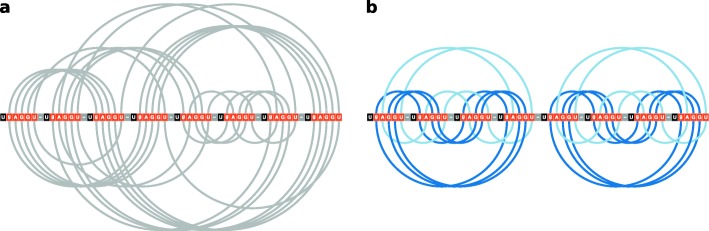
Arc diagram generated by ElTetrado for DNA/RNA hybrid (PDB ID: 1N7A) **a** without and **b** with strand reordering

An example outcome of ElTetrado running with and without the reordering procedure is shown in Fig. 1. The analysed structure of DNA/RNA hybrid (PDB ID: 1N7A) [23] is composed of eight strands which form ten tetrads. ElTetrado called with --no-reorder parameter is not able to classify the tetrads and generates the arc diagram as shown in Fig. 1a. If the parameter is not passed, the reordering procedure is executed. In this case, ElTetrado identifies two regular 5-tetrad quadruplexes assigned to Op category (Fig. 1b). The first one includes four O+ type tetrads (dark blue) and one O- type (light blue) quartet. The second quadruplex has three O+ tetrads and two tetrads in O- group.

Using the reordering procedure, we executed ElTetrado for a set of tetrads and quadruplexes identified in nucleic acid structures deposited in the Protein Data Bank as of 18 December, 2019 (Tables 1, 2, and 3). In this experiment, we were particularly interested in the coverage of various categories in the ONZ taxonomy by bi- and tetramolecular motifs. The analysed dataset contained 328 structures, 148 of which were determined by X-ray crystallography (107 DNAs, 38 RNAs and 3 modified nucleic acids) and 180 by NMR (169 DNAs, 9 RNAs, 2 modified). All X-ray structures in this collection have good resolution. Its minimum value is 0.61 Å, maximum 3.793 Å, and average resolution is 2.148 Å. In the considered structures, ElTetrado identified 1445 tetrads and 452 quadruplexes in total. The analysis showed that most tetrads (namely 1035) in the collection were in O-type classes (O+ type tetrads accounted for 86% of this group, while O- tetrads for 14%). Moreover, ElTetrado found 197 tetrads from N categories (65% of them were in N+, 35% in N-), and 213 from Z (half in half in classes Z+ and Z-). Among quadruplexes, we found 424 regular motifs and 28 irregular ones. The subset of regular quadruplexes contained 312 cases from class O (72% of them were in Op, 16% in Oa, and 12% in Oh), 58 from class N (88% of them were in Na, 12% in Nh), and 54 from class Z (98% of them in Za, 2% in Zh). Among irregular quadruplexes, 46% of them were in Mp and 54% in Mh category. Let us note that among bi- and tetramolecular quadruplexes, none was in negative (-) subcategory. These quadruplexes belonged to positive (+) or hybrid (*) subtypes (Table 3).

**Table 1:** ONZ class coverage by uni-, bi- and tetramolecular tetrads

Tetrads	O+	O-	N+	N-	Z+	Z-	Total
Unimolecular	475	83	104	51	6	8	727
Bimolecular	87	10	25	17	101	98	338
Tetramolecular	332	48	-	-	-	-	380
Total	894	141	129	68	107	106	1445

**Table 2:** ONZM class coverage by unimolecular quadruplexes

	Unimolecular quadruplexes							
	Op	Oa	Oh	Na	Nh	Mp	Mh	Total
+	105	-	12	11	3	8	8	147
-	2	-	-	-	-	-	-	2
*	13	40	23	27	4	5	6	118
Total	120	40	35	38	7	13	14	267

**Table 3:** ONZM class coverage by bi- and tetramolecular quadruplexes

	Bimolecular									Tetramolecular		
	Op	Oa	Oh	Na	Za	Zh	Mh	Total		Op	Oa	Total
+	23	-	-	3	-	-	-	26		49	1	50
-	-	-	-	-	-	-	-	-		-	-	-
*	2	4	2	10	51	1	1	71		30	6	36
Total	25	4	2	13	51	1	1	97		79	7	86

## Conclusions

Recent study of the topology of quadruplex secondary structure bore fruit with new approach to analyse and categorize these motifs [20]. ElTetrado is the first software tool that - apart from identification of tetrads and quadruplexes - can assign them to classes defined in ONZ taxonomy. It can also prepare their unequivocal representation in dot-bracket notation and arc diagram. We believe that by providing an insight into the secondary structure features, ElTetrado complements formalisms proposed for canonical G4s [19]. Thus, our method will contribute to a better understanding of quadruplex architecture and improve its full description.

## Methods

**Fig. 2:**
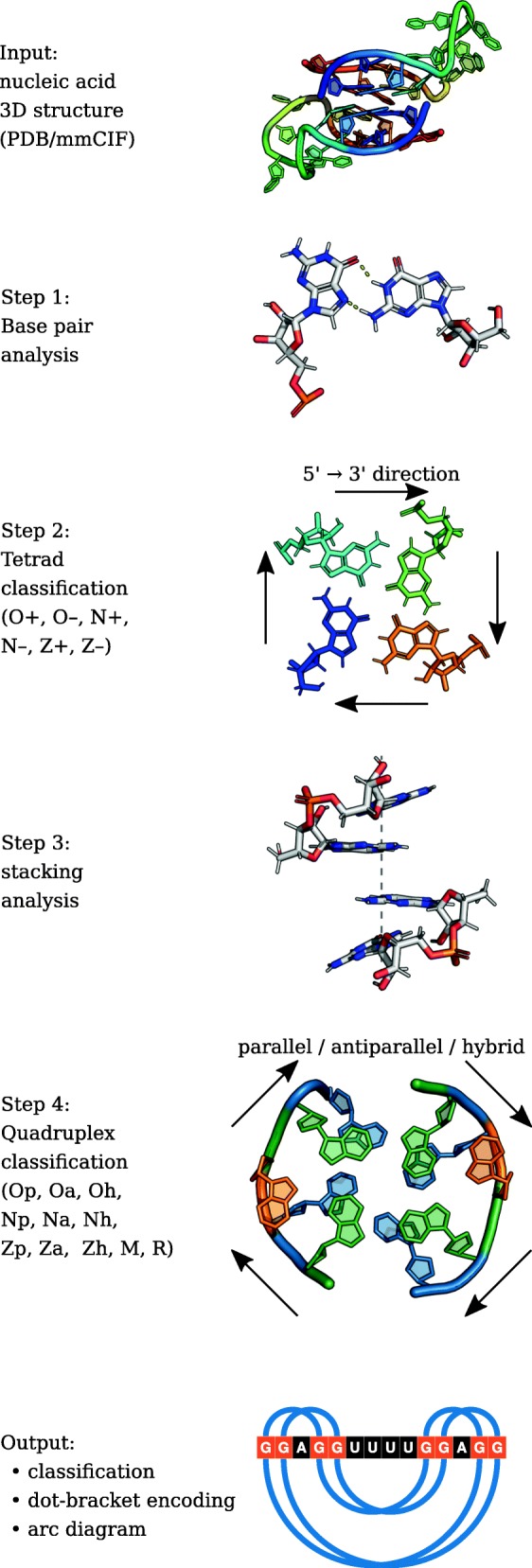
Consecutive steps in the ElTetrado workflow

ElTetrado processes PDB and mmCIF files to identify quadruplexes and their component tetrads in nucleic acid structures (Fig. 2). It applies DSSR [24] to collect the preliminary information about base pairs and stacking. Next, it scans the properties of intra-tetrad interactions to determine tetrad categories within ONZ taxonomy. Once the tetrads are classified, the algorithm identifies quadruplexes. These motifs are next categorized based on the classes of their component quartets and the order of nucleotides in the N-tract. Finally, ElTetrado prepares the dot-bracket representation of the secondary structure of identified quadruplexes. It also applies R4RNA package [25] to create the output arc diagram.

### Tetrad classification procedure

There are six classes of tetrad topologies in ONZ taxonomy: O+, O-, N+, N-, Z+ and Z- [20]. They differ in hydrogen-bonding interactions, i.e., in each class, the other nucleotide residues form base pairs, along the other edges - Watson-Crick, Hoogsteen or Sugar edge.

Let us have four residues building tetrad T. We will mark them with letters A, B, C, D. Suppose that in the sequence, A is the first from 5’-end of these four residues. Assume also that the following base pairs are formed in T: (A,B), (B,C), (C,D), (D,A), where the first residue of the pair binds with the second one along its Watson-Crick edge, while the second with the first along its Hoogsteen or Sugar edge (for example, in pair (A,B), A binds along its Watson-Crick edge, B binds along its Hoogsteen edge). Let us now check in what order the residues are arranged in the nucleotide sequence from 5’ to 3’ end. Depending on this arrangement, tetrad T is assigned to a given category:

- if 5’ - A - B - C - D - 3’ then T ∈ O+
- if 5’ - A - D - C - B - 3’ then T ∈ O-
- if 5’ - A - B - D - C - 3’ then T ∈ N+
- if 5’ - A - C - D - B - 3’ then T ∈ N-
- if 5’ - A - C - B - D - 3’ then T ∈ Z+
- if 5’ - A - D - B - C - 3’ then T ∈ Z-.

A pseudocode of tetrad classification procedure applied in ElTetrado is given in Algorithm 1.


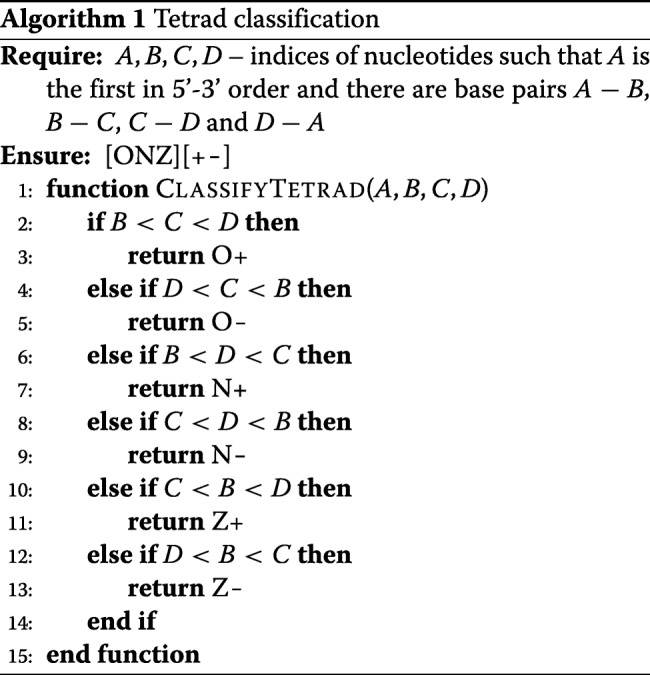


### Quadruplex classification procedure

In the case of quadruplexes, we have distinguished between regular and irregular structures. A regular motif contains tetrads of one general type, i.e. O, N or Z. We pay attention to the order of nucleotides in N-tracts, thus having parallel (p), antiparallel (a) or hybrid (h) motifs (cf. Algorithm 2). This way we define nine classes of regular quadruplexes: Op, Oa, Oh, Np, Na, Nh, Zp, Za, Zh.

Let us have a quadruplex Q composed of three tetrads, T _*i*_(A _*i*_-B _*i*_-C _*i*_-D _*i*_), T _*j*_(A _*j*_-B _*j*_-C _*j*_-D _*j*_) and T _*k*_(A _*k*_-B _*k*_-C _*k*_-D _*k*_), all of them in the O+ category. Quadruplex Q is assigned to a given category if the following order of residues is preserved in the nucleic acid strand:

- if 5’ - A _*i*_*A*_*j*_*A*_*k*_ - B _*i*_*B*_*j*_*B*_*k*_ - C _*i*_*C*_*j*_*C*_*k*_ - D _*i*_*D*_*j*_*D*_*k*_ - 3’ then Q ∈ Op
- if two tracts have different order than the other two (for example, 5’ - A _*i*_*A*_*j*_*A*_*k*_ - B _*k*_*B*_*j*_*B*_*i*_ - C _*i*_*C*_*j*_*C*_*k*_ - D _*k*_*D*_*j*_*D*_*i*_ - 3’) then Q ∈ Oa
- if one tract has a different order than the other three (for example, 5’ - A _*i*_*A*_*j*_*A*_*k*_ - B _*k*_*B*_*j*_*B*_*i*_ - C _*k*_*C*_*j*_*C*_*i*_ - D _*k*_*D*_*j*_*D*_*i*_ - 3’) then Q ∈ Oh.

If a quadruplex is built from tetrads of different types, it is assigned to category M (mixed) that collects irregular motifs. Regular and irregular categories of quadruplexes can be further divided into positive (+), negative (-) and hybrid (*) subcategories, depending on the + and - signs in the classification of quadruplex-building tetrads. For example, if a parallel quadruplex contains only O+ tetrads it is assigned to Op+ group, if it is built of O- tetrads only we assign it to class Op-, and if it contains a mixture of O+ and O- tetrads then it belongs to Op* subcategory.

For the purposes of quadruplexes with unclassified tetrads we have also introduced the R class (remaining ones) [20]. However, recent computational experiments have not shown the existence of motifs that could belong to this class.

Algorithm 3 presents the quadruplex classification procedure executed in ElTetrado.


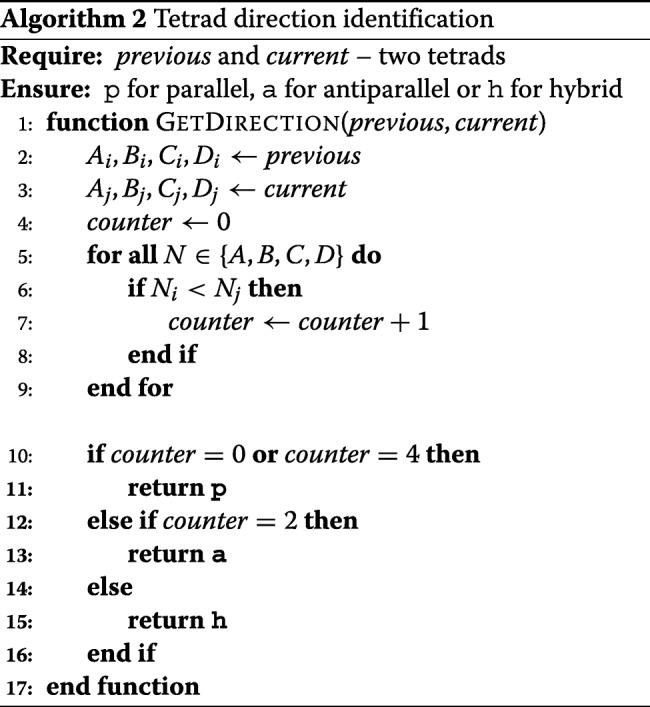
 
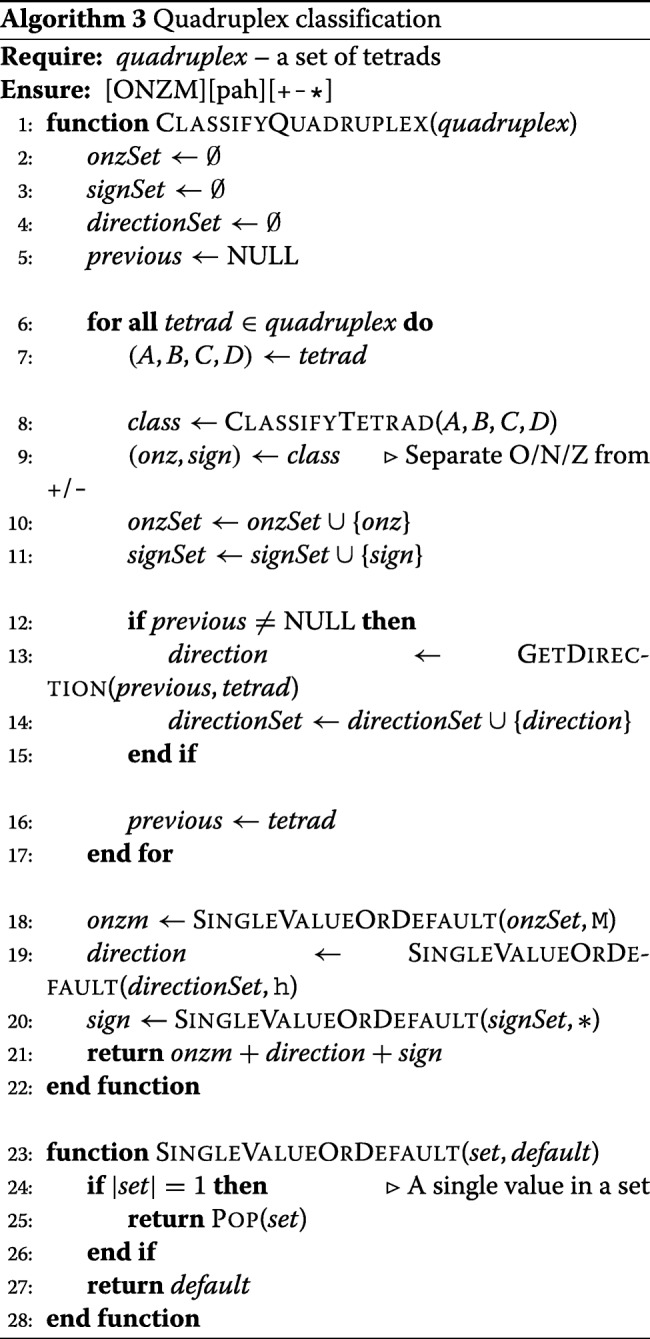


### Running ElTetrado

ElTetrado is a standalone application implemented in Python 3, using its standard libraries. Therefore it works under all common operating systems. In order to start working with the program, the users need to download it onto the local drive from https://github.com/tzok/eltetrado.

We recommend that, apart from ElTetrado, the users should download the DSSR binary [24] and place it in the same local directory. DSSR is utilized for the preliminary analysis of base pairs in the input 3D structure. Its local installation allows the users to control DSSR execution. For example, one can decide to pass --symmetry parameter to x3dna-dssr binary when dealing with X-ray structures, which is necessary for some quadruplexes. Alternatively, if the users do not want to have a local version of DSSR binary, they can obtain the DSSR output in JSON format from any place and use them as input data for ElTetrado (with --dssr-json parameter).

ElTetrado is started from the command line. The users enter the program name and either --pdb followed by an input file name (the file should be in PDB or mmCIF format), or --dssr-json followed by a path to JSON file generated by DSSR, or both switches at once. Optionally, users can pass the following input parameter(s) that allow controlling ElTetrado operation mode: --stacking-mismatchThis parameter can take one of three values: 0, 1, 2 (default). It controls the algorithm’s tolerance of mismatches when analyzing tetrad stacking. If stacking-mismatch equals 0, then the ideal geometry is enforced. It means that exactly all 4 pairs of nucleotides should be stacked for two tetrads to be considered the quadruplex or N4-helix. If stacking-mismatch equals 2 (default value), then the room is left for some flexibility and imperfect stacking. --relaxed-stem-definitionIf this parameter is passed, ElTetrado treats sequentially neighbouring tetrads as part of the same quadruplex, regardless of their mutual stacking interactions. --strictIf this parameter is set, ElTetrado looks for cis Watson-Crick–Hoogsteen base pairs only when looking for tetrads. By default, this mode is switched off what allows ElTetrado to find tetrads linked by base pairs of different types. --no-reorderThe ONZ classification is based on strand direction from 5’ to 3’. When multiple strands contribute to build the quadruplex, their order influences the assigned class. Therefore, ElTetrado checks all permutations of chain orders in bi- and tetramolecular quadruplexes before classifying them. If this parameter is set, chain order remains the same as in the input file and bi- and tetramolecular quadruplexes are not classified. --complete-2dRunning ElTetrado with this parameter causes that in the arc diagram, apart from the quadruplex, canonical base pairs occurring in the remaining part of the structure will be represented. This option allows seeing the quadruplex in the context of the entire structure.

The arcs in the arc diagram are assigned colors depending on the category of represented tetrads. By default, class O is blue, class N is green, and class Z is orange. Positive (+) and negative (-) subcategories are distinguished by light and dark shades of the same color, respectively. For example, O+ is dark blue, O- is light blue in the diagram. Unclassified tetrads are colored grey. Canonical base pairs that do not belong to the quadruplex are represented by black arcs. However, the users can define in the configuration file any set of colors for annotating ONZ classes in the diagram.

### Output description

Output data includes the following information about quadruplexes identified in the input structure: (i) quadruplex category in ONZ-based taxonomy, (ii) the list of tetrads that compose the motif with their ONZ classification, (iii) quadruplex secondary structure in dot-bracket notation, (iv) arc diagram representing quadruplex secondary structure. Example output data for the input PDB structure of dimeric RNA quadruplex (PDB id: 1MY9) [26] is presented on the bottom panel of Fig. 2.

## Availability and requirements

**Project name:** ELTetrado **Project home page:**https://github.com/tzok/eltetrado**Operating system(s):** Platform independent **Programming language:** Python 3 **Other requirements:** DSSR binary **License:** MIT **Any restrictions to use by non-academics:** no restrictions

## Data Availability

All the data and materials are available at the project home page.

## Abbreviations

G4: Guanine quadruplex
mmCIF: Macromolecular crystallographic information file
NMR: Nuclear magnetic resonance
ONZ: O-shaped, N-shaped, Z-shaped
PDB: Protein data bank
